# Prediction of hypericin content in *Hypericum perforatum* L. in different ecological habitat using artificial neural networks

**DOI:** 10.1186/s13007-021-00710-z

**Published:** 2021-01-26

**Authors:** Maryam Saffariha, Ali Jahani, Reza Jahani, Sajid Latif

**Affiliations:** 1 Department of Reclamation of Arid and Mountainous Regions, College of Natural Resources, University of Tehran, Tehran, Iran; 2 Faculty of Natural Environment and Biodiversity Department, College of Environment and Research Center of Environment and Sustainable Development, Standard Square, Karaj, Iran; 3 Department of Pharmacology and Toxicology, School of Pharmacy, Shahid Beheshti University of Medical Sciences, Tehran, Iran; 4 Graham Centre of Agricultural Innovation, Charles Sturt University, Wagga Wagga, Australia

**Keywords:** Artificial neural network, Ecological modeling, Graphical user interface, Hypericin, *Hypericum perforatum*

## Abstract

**Background:**

*Hypericum* is an important genus in the family *Hypericaceae*, which includes 484 species. This genus has been grown in temperate regions and used for treating wounds, eczema and burns. The aim of this study was to predict the content of hypericin in *Hypericum perforatum* in varied ecological and phenological conditions of habitat using artificial neural network techniques [MLP (Multi-Layer Perceptron), RBF (Radial Basis Function) and SVM (Support Vector Machine)].

**Results:**

According to the results, the MLP model (R^2^ = 0.87) had an advantage over RBF (R^2^ = 0.8) and SVM (R^2^ = 0.54) models and it was relatively accurate in predicting hypericin content in *H. perforatum* based on the ecological conditions of site including soil types, its characteristics and plant phenological stages of habitat. The results of sensitivity analysis revealed that phenological stages, hill aspects, total nitrogen, altitude and organic carbon are the most influential factors that have an integral effect on the content of hypericin.

**Conclusions:**

The designed graphical user interface will help pharmacognosist, manufacturers and producers of medicinal plants and so on to run the MLP model on new data to easily discover the content of hypericin in *H. perforatum* by entering ecological conditions of site, soil characteristics and plant phenological stages.

## Background

*Hypericum perforatum* L. is among the most important species of the genus *Hypericum* which is being studied for several therapeutic purposes including skin wounds and burns [15]. Previous literature has reported its role as neuroprotective [15], antidepressant [15], antioxidant [9], hepatoprotective [15], antimicrobial, and antiviral [9]. Among the known constituents of *H. perforatum*, hypericin and flavonoids attribute to several pharmacological effects of *H. perforatum*. Therefore it remain imperative to develop methods and/or tools accurately identify and predict the concentration of hypericin in *H. perforatum* [15].

It is likely that the content of hypericin content in plant tissue is impacted by physiological and environmental factors including climate, topography, vegetative stage, epigenetic similar to other related secondary metabolites [15, 43]. However, there is limited evidence available in the literature reporting association of hypericin contents with biotic and abiotic factors [15]. It is interesting to study association of various ecological factors affecting biosynthesis and tissue localization of secondary metabolites in regards to harvesting maximum yield of biologically active constituents from plant extracts. Previous studies demonstrated a strong correlation between the altitude and hypericin content in plant extracts, considering the intensity of sunlight and temperature as primary factors [15, 43]. For instance, Asghari et al. [1] reported significant effect of the ecological conditions mainly altitude of 300, 600 and 1200 m above the sea level on the hypericin content in different highlands of Golestan National Park of Iran. Saffariha et al. [15] studied essential oil yield of *Salvia limbata* L. (Sage) at three different altitudes and reported a significant difference in the amount of essential oil at 2500 m (highest altitude) altitude compared to other sites.

Furthermore, sample harvesting, extraction and analytical processing presents a wider range of discrimination in regards to the accurate quantification of hypericin in *H. perforatum* [15]. High-performance liquid chromatography (HPLC) technique with different type of detectors such as diode array, fluorescence, and mass detector have been employed for robust identification and determination of hypericin in crude extracts of *H. perforatum* [7]. Although these techniques have high resolution to detect metabolites at very low concentration but they present some limitation including instrument cost, laboratory space and require large volumes of mobile phase and personal skills. In addition, it often require development of complex time consuming workflows comprising of sample collection, extraction purification and analytical method development, data collection and processing and statistical analysis [15]. Therefore a relatively simple, robust and eco-friendly tool are required for prediction of secondary metabolites including hypericin.

Artificial neural network (ANN) modeling technique has been designed based on the human brain functioning with a variety of mathematical functions to enhance the ability of the model for accurate prediction. Recently, artificial neural network techniques such as Multi-Layer Perceptron (MLP) neural network, Radial Basis Function (RBF) and Support Vector Machine (SVM) as nonlinear and dynamic modeling techniques are utilized in ecological sciences for development of accurate prediction models [15]. For example, Saffariha et al. [15] used artificial neural network modeling to predict seed germination of *Salvia limbata* L. under ecological stresses and the reported accurate association of observed data with predicted data. This unique quantitative modeling and artificial neural network approach are essential to deal with ecological and biological phenomena such as the prediction of hypericin content in *H. perforatum*.

In this research, we aimed to predict the concentration of hypericin in *H. perforatum* in a range of ecological and phenological conditions comparing artificial neural network modeling techniques such as MLP, RBF and SVM. This study will report the most accurate model predicting hypericin concentration as impacted by ecological and phenological factors. Finally, the Graphical User Interface (GUI) tool as an environmental decision support system will be designed for ecologists to predict the amount of hypericin in *H. perforatum*.

## Materials and methods

### Study area and sampling

This study was performed in the south of Alborz Mountain protected area, located in Alborz province of Iran (35° 44′ N to 36° 35′ N and 51° 00′ E to 51° 36′ E). Alborz protected area is one of the main habitats of *H. perforatum* in Iran. *H. perforatum* grows at an altitude range from 1000 to 4000 m in different ecological conditions.

To cover a variety of ecological conditions in the area, we collected 100 plant samples along with 15 linear transects (the length of each transect equals to 1000 m) at different altitudes. Plant sampling was carried out for every 200 m altitude (from 1000 m) by a linear transect with a length of 1000 m started from the beginning of the altitude class. Each plant (*H. perforatum*) that touched the transect line was sampled making a total of 100 samples. The aerial parts of *H. perforatum* were collected at three stages namely; (1) vegetative, (2) flowering, and (3) seed ripening stages of plants over the period of 6 months in growing season. Since the chemical composition varies in different tissue of the plant, we used composite sample by mixing all tissue at equal proportion for all samples.

Soil features such as organic carbon (%), total nitrogen (%), absorbable phosphor (ppm), absorbable potassium (ppm), sand (%), silt (%), clay (%), electrical conductivity (EC) and acidity (pH) were measured at each sample point. Landform characteristics of site including altitude (m), slope (%) and hill aspect [four aspects including (1) north, (2) east, (3) south and (4) west], in each sample point were recorded.

Plant materials were air-dried in shadow at room temperature for a week and then were ground in a grinder with a 2 mm mesh size. Voucher specimens were transformed at the Herbarium of Medicinal Plants and Drugs Research Institute, Shahid Beheshti University, Tehran, Iran.

### Extraction of *Hypericum perforatum*

Plant extraction was performed as described by Ramezani et al. [15] with some modifications. In order to remove chlorophylls and unwanted nonpolar constituent, the ground aerial parts of *H. perforatum* (100 g) were placed in a 1000 mL volumetric flask and made up to volume with chloroform (Merck, Germany). The mixture was placed on a stirrer at room temperature overnight. Then the chloroform fraction was filtered off through Whatman filter paper and the supernatant containing chlorophyll and unwanted compounds was discarded. This procedure was repeated three times until colorless supernatant was obtained. The residual chloroform was evaporated using nitrogen gas at 40 °C. Then the dried residue was dissolved in 1 L of acetone and the solution was sonicated for 20 min in an ultrasonic bath (Elma S30H, Germany) before allowing it to stir overnight. This procedure was repeated for 4 consecutive days and the acetone extracts were combined and evaporated on a rotary evaporator and dried weight was recorded. Finally, the residue was reconstituted in methanol (Merck, Germany) at the concentration of 10 mg/mL and the sample was filtered through a 0.45 µm PFTE filter (Gelman Sciences) prior to HPLC analysis.

### HPLC analysis of hypericin

**Fig. 1:**
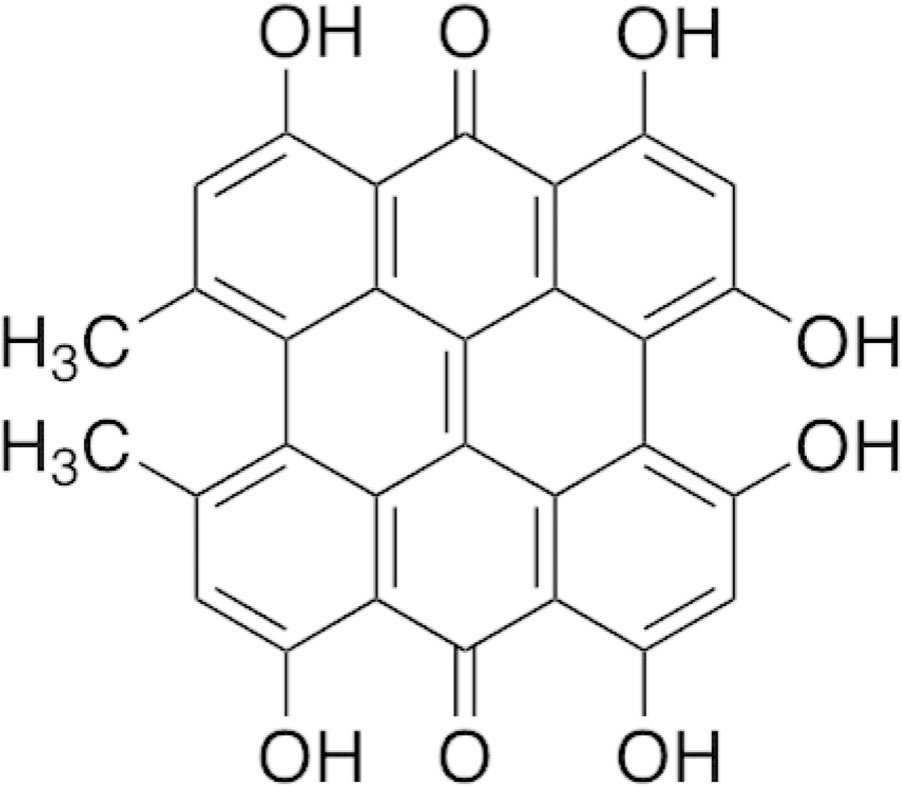
Chemical structures of the hypericin

All chemicals and reagents were of analytical grade. Acetonitrile, methanol and phosphoric acid were purchased from Merck (Darmstadt, Germany). HPLC grade hypericin (Fig. 1) was obtained from Sigma-Aldrich (St. Louis, MO). The HPLC analysis of hypericin in *H. perforatum* was conducted on an Agilent Series 1200 system (Palo Alto, CA, USA) equipped with a quaternary pump, an online degasser and an Agilent diode array detector which was set on 590 nm. Hypericin separation was performed on a Diazem-phenyl™ (Metachem Technologies, 5 µm, 250 × 4.6 mm) column that was connected to a Diazem-phenyl™ guard column cartridge. The mixture of acetonitrile, methanol, water and phosphoric acid (48:40:10:2) with the flow rate of 1 mL/min was used as a mobile phase. The column temperature, run time and injection volume were set on 30 °C, 15 min and 20 µL, respectively [15]. The stock solution of hypericin was gravimetrically prepared by dissolving 1 mg hypericin (95% purity) in 10 mL methanol and the calibration standard working solutions were freshly prepared by dilution of the stock solution with methanol. In order to confirm the presence of hypericin in the analyzed samples, retention times and the spectra of the peaks in the chromatogram were compared to those of hypericin standard solution. Each sample was analyzed in triplicate and the hypericin concentration was expressed as mg/g dry mass.

### Modeling process

Considering the aim of this research, *H. perforatum* samples were collected in different land forms, soils and phenological stages in Alborz protected area to have diversity in hypericin content in samples.

The relation of environmental variables varies based on ecosystem conditions from linear to nonlinear correlations. Therefore, the classic modeling methods such as linear regression result in less accurate predictions in comparison with artificial neural network (ANN) models. The ANN function in MATLAB 2018, was used to design the structure of three models.

### Multi-layer perceptron neural network (MLP)

We applied the MLP model in a process that includes elements named as neurons [12]. Since we aimed to evaluate the most precise model, the number of transfer functions, neurons and hidden layers, were carefully modified. To optimize the model structure and maximize the accuracy of outputs, the number of hidden layers and neurons were determined by trial and error and recursive testing and comparison [15]. The transfer function was also selected based on trial and errors to find the most precise model and upgrading the outputs (refer to Demuth and Beale [5]). Based on the literature review, 13 effective variables were used to predict hypericin content in *H. perforatum*. In this method, input variables (land form, phenological stages and soil characteristics) and output variables (hypericin content) were tested against each sample to train MLP. As input variables, land form variables are: altitude (m), land slope (%) and hill aspect (four geographical hill aspect consist of north, east, south and west); phenological stages are: (1) vegetative, (2) flowering, and (3) seed ripening and soil characteristics include: organic carbon (%), total nitrogen (%), absorbable phosphor (ppm), absorbable potassium (ppm), sand (%), silt (%), clay (%), electrical conductivity (EC) and acidity (pH).

The main role of the transfer function is summarizing the weighted variables to achieve the most accurate model outputs [15]. In the modeling process, 60 percent of samples (n = 60) were applied in the training step. The remaining 40 samples were divided equally (20, 20) in two data sets for validation and testing. In MLP training, the weights (w) of the ith variable (x) in jth neuron were defined to calculate the output of jth neuron on the kth hidden layer (${\text{net}}_{{\text{j}}}^{{\text{k}}}$) by Eq. 1.

1 $${\text{net}}_{{\text{j}}}^{{\text{k}}} = \mathop \sum \limits_{{{\text{i}} = 0}}^{{\text{n}}} {\text{w}}_{{{\text{ji}}}} {\text{x}}_{{{\text{ji}}}} .$$

The output of Eq. 1 is defined as the input of a transfer function (∫) in Eq. 2. The different transfer functions are tested to find out the most precise one in the generation of accurate output.

2 $${\text{Y}}_{{{\text{net}}}} = \smallint {\text{net}}_{{\text{j}}} .$$

To justify the most appropriate weights of neurons and layers, we used back propagation method in Eq. 3 for calculation of errors between predicted and target content of hypericin. In Eq. 3, “E” is the sum of squared errors, w_ji_ represents the weight of ith neuron in jth hidden layer, and γ is the learning rate which is determined by a crisp value.

3 $${\text{w}}_{{{\text{ji}}}}^{{\text{t}}} = {\text{w}}_{{{\text{ji}}}}^{{{\text{t}} - 1}} + \left( { -\upgamma \frac{{\partial {\text{E}}^{{\text{t}}} }}{{\partial {\text{w}}_{{{\text{ji}}}}^{{\text{t}}} }}} \right).$$

### Radial basis function neural network (RBFNN)

The RBFNNs are designed in a structure of neurons and layers like MLP. The most frequently used radial basis function is the Gaussian function [15] and the center of circular classifiers, in multi-dimensional space is calculated by Eq. 4.

4 $$R_{j} \left( x \right) = \exp \left( {\frac{{\left\| {x - a_{j} } \right\|^{2} }}{{2\sigma^{2} }}} \right).$$

In Eq. (4), R_j_(x) is the radial basis function (RBF), ||x − a_j_|| is the determined Euclidean distance between the total of a_j_ (RBF function center), x is the (input vector or variables), and σ is a positive real number.

In the last step, the network outputs or predicted hypericin content are calculated with Eq. (5):

5 $$y_{k} = \mathop \sum \limits_{j = 1}^{m} w_{jk} R_{j} \left( x \right) + b_{j} .$$

In Eq. (5), w_ik_ is the weights of neurons, j is the number of each node in the hidden layer, m is the number of neurons, and b_j_ is the bias [15].

### Support vector machine (SVM)

SVM is a classifier which is developing the margins around the boundaries of classification. SVM model Eq. (6) uses input variables in the structure of a kernel function (Eq. 7).

6 $$y\left( x \right) = \mathop \sum \limits_{i = 1}^{n} \alpha_{i} K\left( {x_{i} ,x_{j} } \right) + b.$$

The kernel function is defined as Eq. 7. The parameters of Eq. (7) are x_i_ and x_j_ is the samples and γ is the kernel parameter.

7 $$K\left( {x_{i} ,x_{j} } \right) = \exp \left( { - \gamma \left\| {x_{i} - x_{j} } \right\|^{2} } \right).$$

The kernel function parameters are x_i_ and x_j_ are the samples and γ is the kernel parameter.

The weights of the network are optimized by minimizing the errors of the SVM network (Eq. 8) in prediction of output. In Eq. (8), the parameters are Σξ_i_ is the training errors, 1/2||w||^2^ is the margin, and C is the tuning parameter.

8 $$\frac{1}{2}\left\| {w^{2} } \right\| + C\mathop \sum \limits_{i = 1}^{n} \xi_{i} .$$

### Accuracy assessment of models

The models’ performance was tested using the test data set by the main statistical indicators which were formulated in Eqs. 9 to 12. In these equations, $y_{i}$ and $\hat{y}_{i}$ is the targets and network outputs, respectively, $\overline{y}_{i}$ is the mean of target values, and N is the number of samples.

9 $$MSE = \frac{{\mathop \sum \nolimits_{i = 1}^{n} \left( {y_{i} - \hat{y}_{i} } \right)^{2} }}{n},$$

10 $$RMSE = \sqrt {\frac{{\mathop \sum \nolimits_{i = 1}^{n} \left( {y_{i} - \hat{y}_{i} } \right)^{2} }}{n}} ,$$

11 $$MAE = \frac{{\mathop \sum \nolimits_{i = 1}^{n} \left| {y_{i} - \hat{y}_{i} } \right|}}{n},$$

12 $$R^{2} = \frac{{\mathop \sum \nolimits_{i = 1}^{n} \left( {\hat{y}_{i} - \overline{y}_{i} } \right)^{2} }}{{\mathop \sum \nolimits_{i = 1}^{n} \left( {y_{i} - \overline{y}_{i} } \right)^{2} }}.$$

### Sensitivity analysis

In three developed models, each variable influences the outputs of the model with a specific value. To quantify and prioritize the value of variables, influencing the hypericin content prediction of the *H. perforatum* samples, we performed a sensitivity analysis for the optimal model. In the sensitivity analysis, we created a data set for each variable in which the target variable was changed in the range of standard deviations. Other variables were fixed at the value of the average. Then, the standard deviation of outputs for each variable changes was measured as model sensitivity for that variable.

### Graphical user interface tool

A Graphical User Interface (GUI), as an Environmental Decision Support System (EDSS) tool, was designed to run the most accurate hypericin model on new samples of *H. perforatum* to represent a situation where it is required to quantify the hypericin content. GUI is a user-friendly tool to predict the hypericin content in plant samples only by entering the ecological conditions of site, land form, soil characteristics and plant phenological stages.

## Results

### Prediction of the MLP performance

**Fig. 2:**
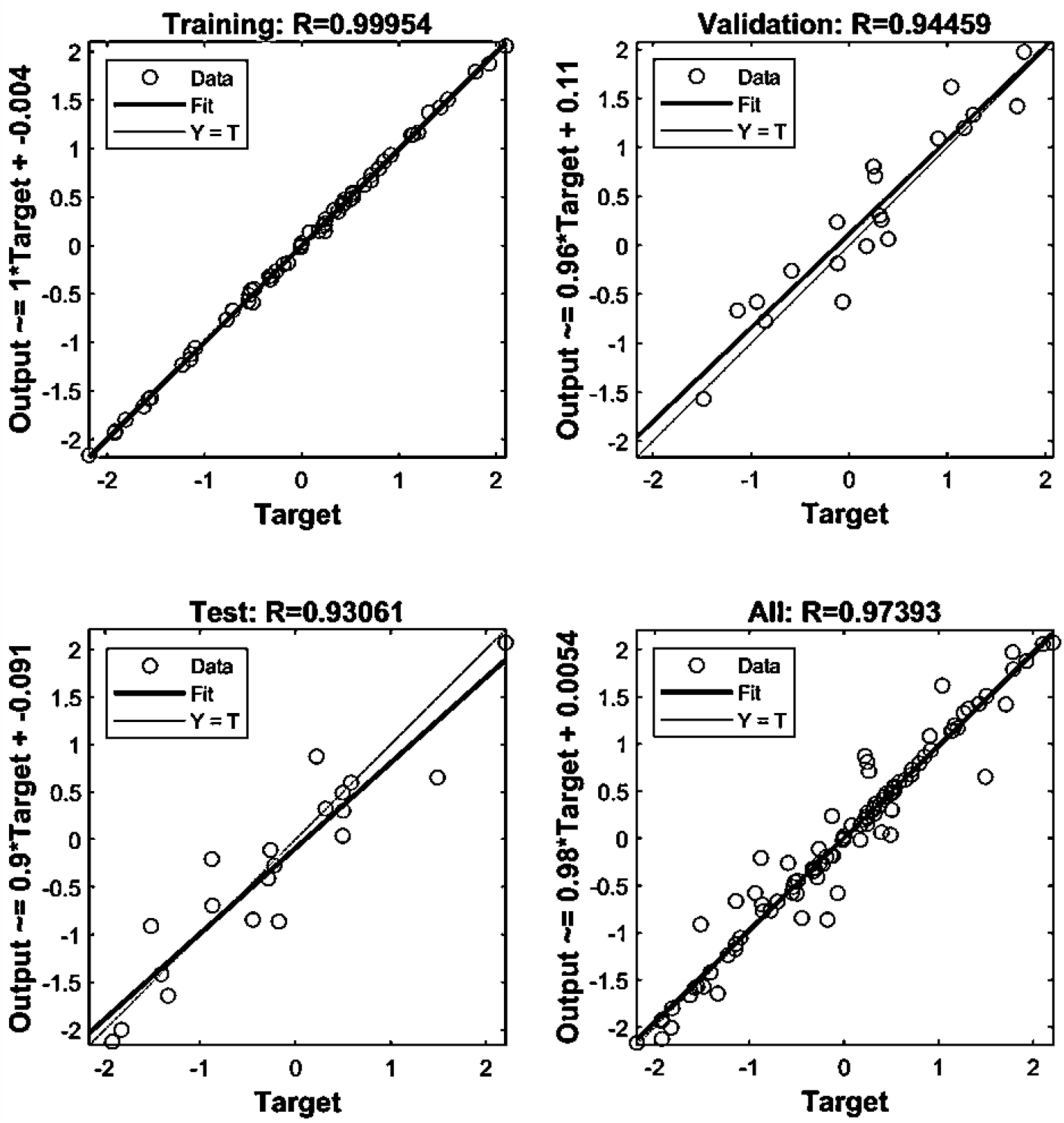
Scatter plots of MLP outputs versus targets values

**Fig. 3:**
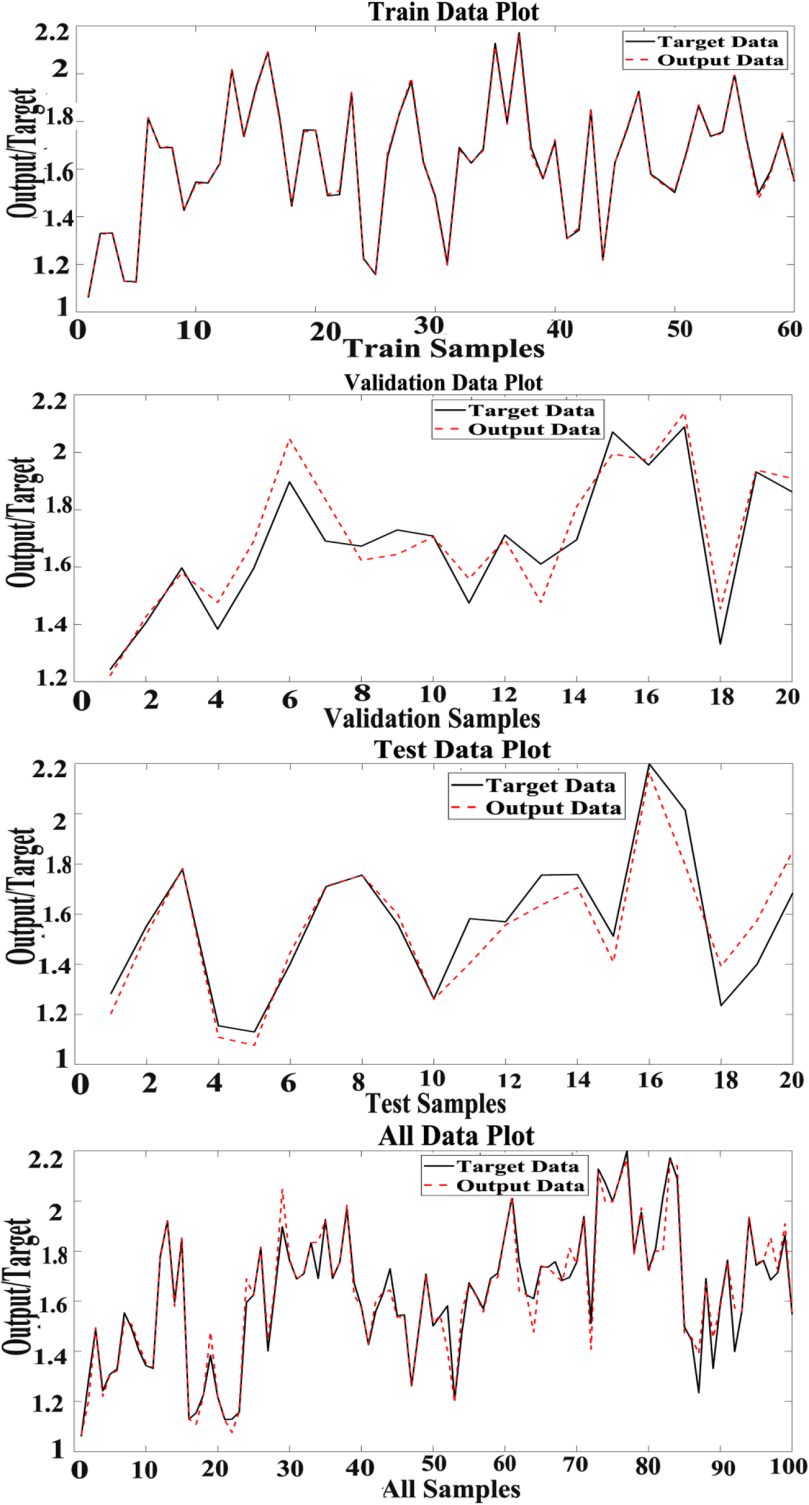
The outputs of MLP and target values of the hypericin content in data sets

Present study utilized three datasets for optimizing the MLP model. We observed that optimizing hidden layers, the number of neurons, active function and training generate relatively more accurate prediction using MLP. The structure of 13-26-1 has been the most accurate for MLP to predict hypericin content in *H. perforatom* extracts as measured of R^2^ values (Table 1). The MLP encompasses 13 variables as inputs, 26 neurons and one variable as the output. Correlation between target and output values are presented in Fig. 2 illustrating MLP outputs versus targets values of the hypericin for training, validation, test, and total data. The coefficient (R^2^) value demonstrate a strong association between MLP outputs and target values (Fig. 3).

**Table 1:** The results of parameters tuning in MLP structure

Activation function	Training function	Structure	Test set				Training Data			
			R^2^	MSE	RMSE	MAE	R^2^	MSE	RMSE	MAE
Logsig-Purelin	LM	13–26-1	0.87	0.15	0.39	0.29	0.99	0.00	0.03	0.02

### Prediction performance of RBFNN

In the training process, there were two main factors including the spreads of RBFs and the number of neurons that were optimized. Decreasing the network error with RBFNN factor values was the purpose of the training step. Thus, to obtain the best performance of RBF, the number of neurons was 48, and the spread of RBFs was 50. The best results of RBF in training and test datasets are represented in Table 2. The values of R^2^ in training and test datasets have been shown the best structure (Table 2). The optimized RBFNN structure is 13-48-1 with 13 variables as inputs, 48 neurons in the hidden layer with Gaussian transfer function, and one neuron.

**Table 2:** The results of parameters (spread and neurons) tuning in RBFNN structure

Model	Spread	Neurons	Test set				Training Data			
			R^2^	MSE	RMSE	MAE	R^2^	MSE	RMSE	MAE
RBF	50	48	0.8	0.16	0.62	0.35	0.98	0.02	0.14	0.12

**Fig. 4:**
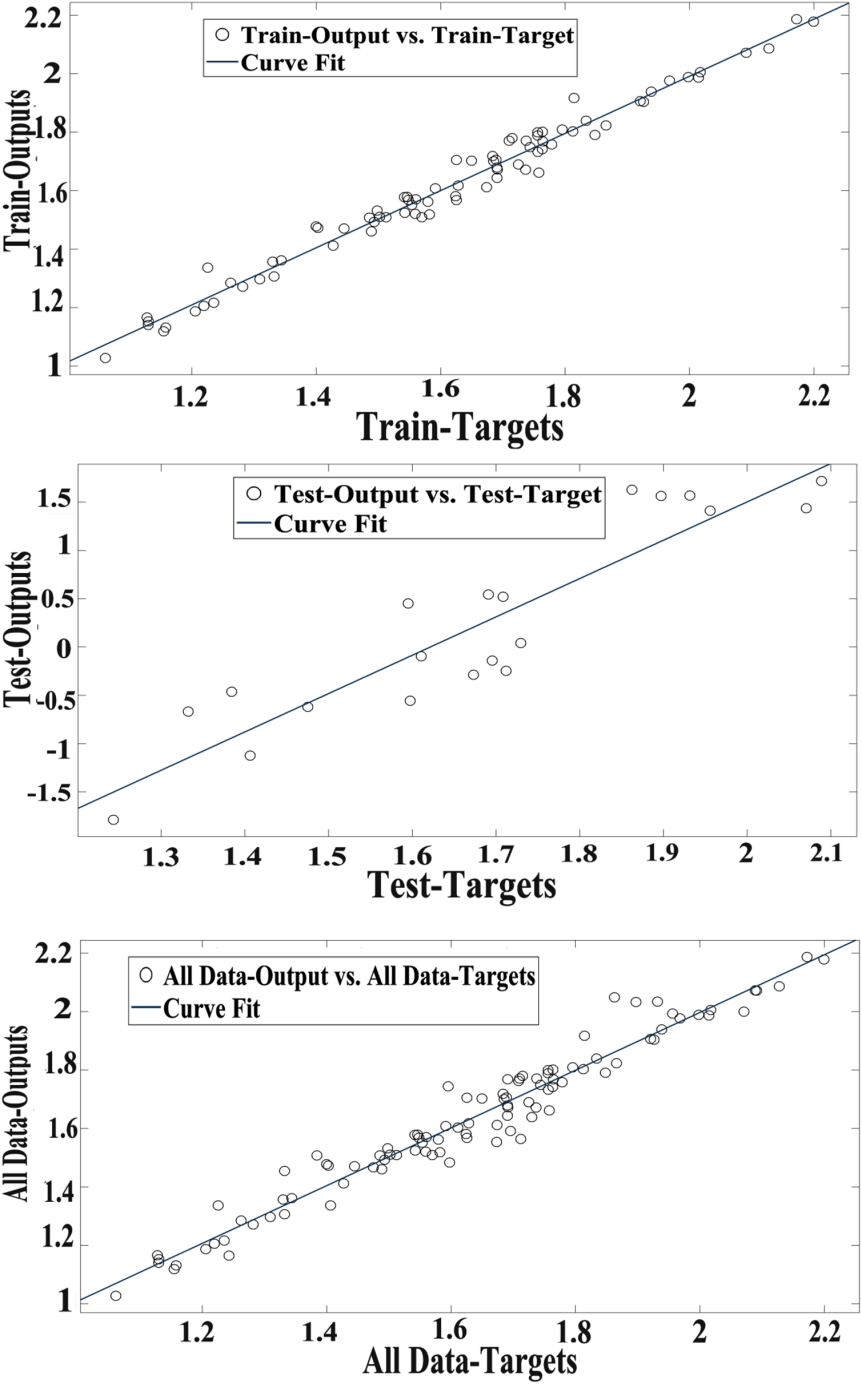
Scatter plots of RBF outputs versus targets values in data sets

Scatter plot of RBFNN outputs versus targets values of the RBF for training, test, and total data are illustrated in Fig. 4.

**Fig. 5:**
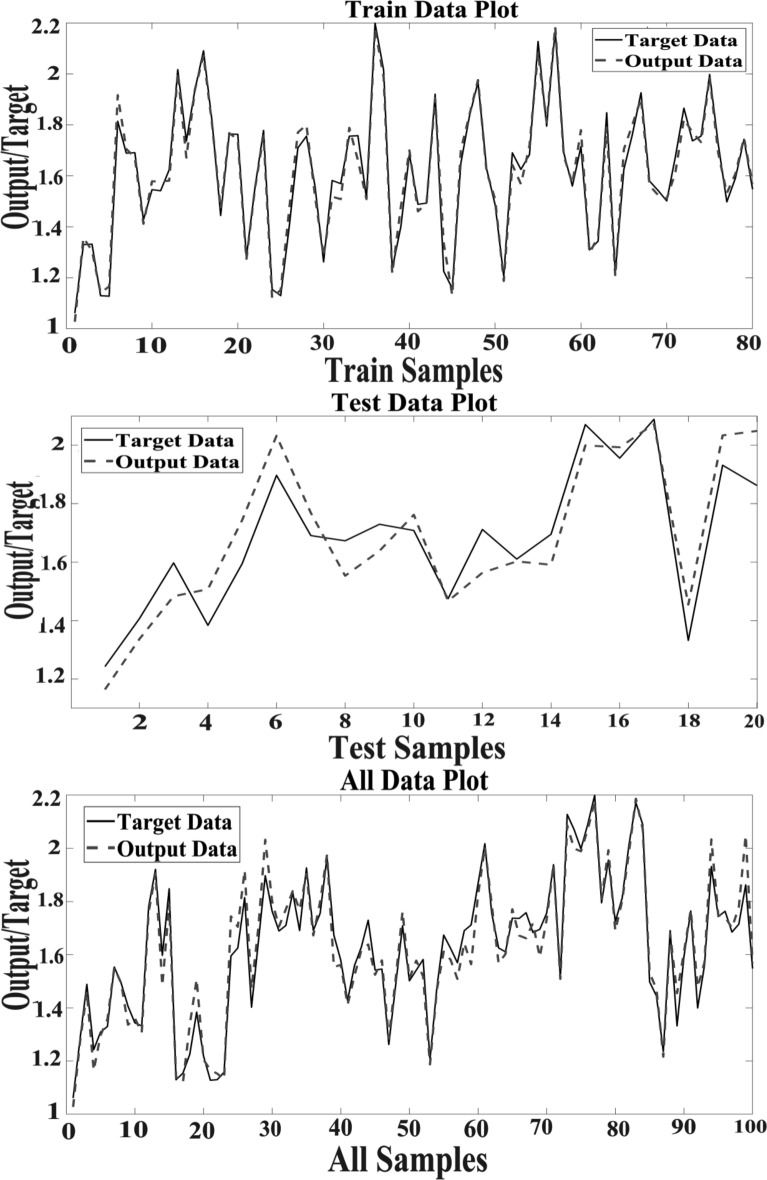
The outputs of RBF and target values of the hypericin volume in data sets

Real (target) and simulated (output) values of RBF in the datasets have been compared and a considerable and defined agreement between values have been represented (Fig. 5).

### Prediction performance of SVM

To determine the number of support vectors, we utilized the value of the parameter ε which is directly related to the vectors [15]. SVM regression that has the Gaussian function includes bell-shaped curves in which the width is determined by value of γ. In this study, when the value of γ is lower, it increases over-fitting. As a result, it was be necessary to obtain the highest accuracy and avoid overtraining. In the SVM model, we were looking for simple curves in which the value of the parameter C helps to achieve this result. In other words, C, ε, and γ factors in SVM regression to obtain hypericin were explained. The most suitable SVM factors and prediction accuracies for SVM regression of the train and test data have been listed in Table 3.

**Table 3:** The value of parameters (ε, C, and γ) tuning in SVM regression structure

Ε	C	Test set				Training data			
		R^2^	MSE	RMSE	MAE	R^2^	MSE	RMSE	MAE
0.0002	995.2	0.54	0.03	0.17	0.14	0.9	0.01	0.08	0.06

According to the values of R^2^ in training and test datasets, the best ε value is 0.0002, and C value is 995.2. Other models with different ε, C, represent over-fitting and under-fitting in models.

**Fig. 6:**
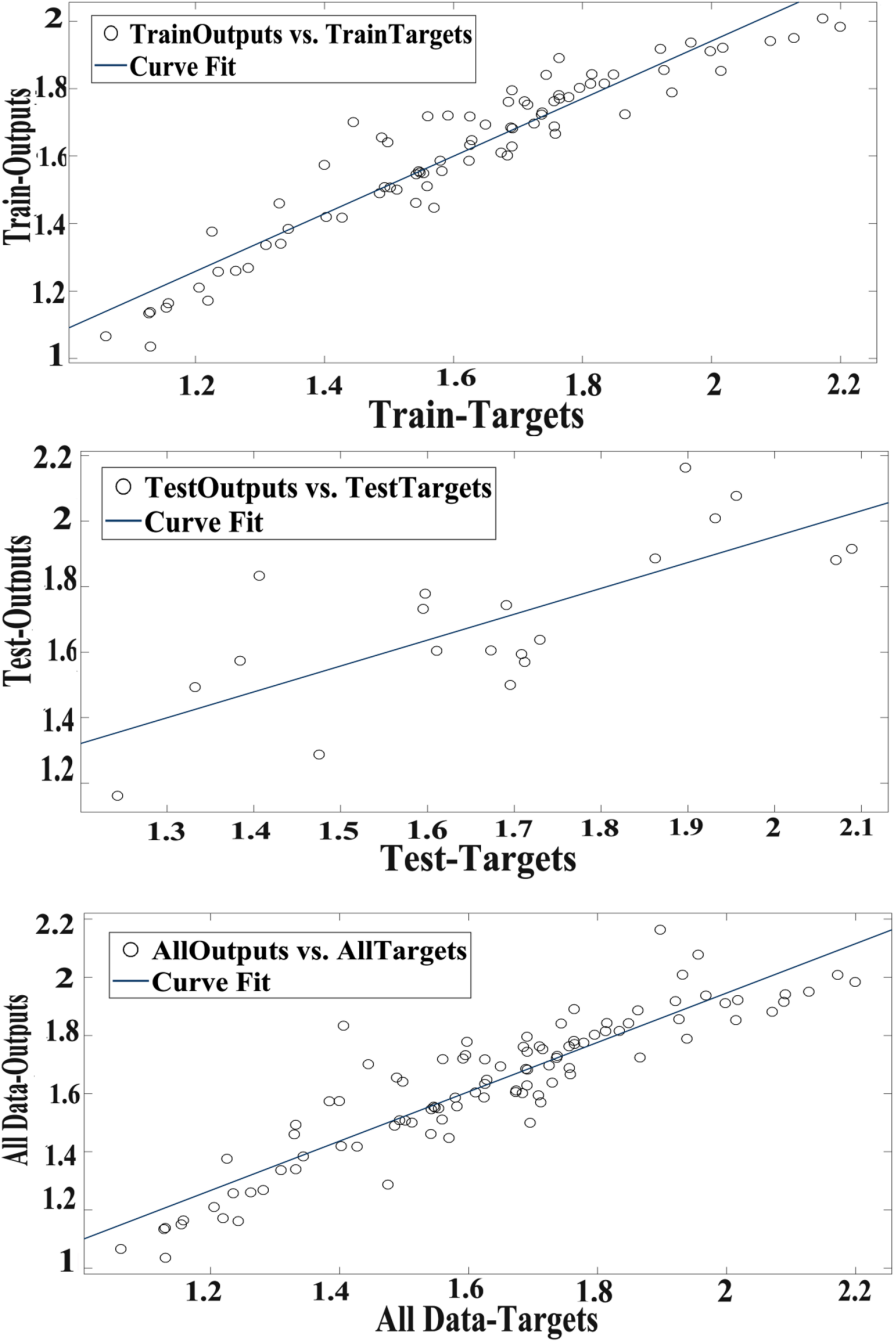
Scatter plots of SVM outputs versus targets values in data sets

Figure 6 illustrates the scatter plot of SVM outputs via target values of the hypericin for training, test, and total data. Coefficient (R^2^) has confirmed a strong correlation between the SVM outputs and targets values. Some simple linear regressions that have been validated are observed in Fig. 6.

**Fig. 7:**
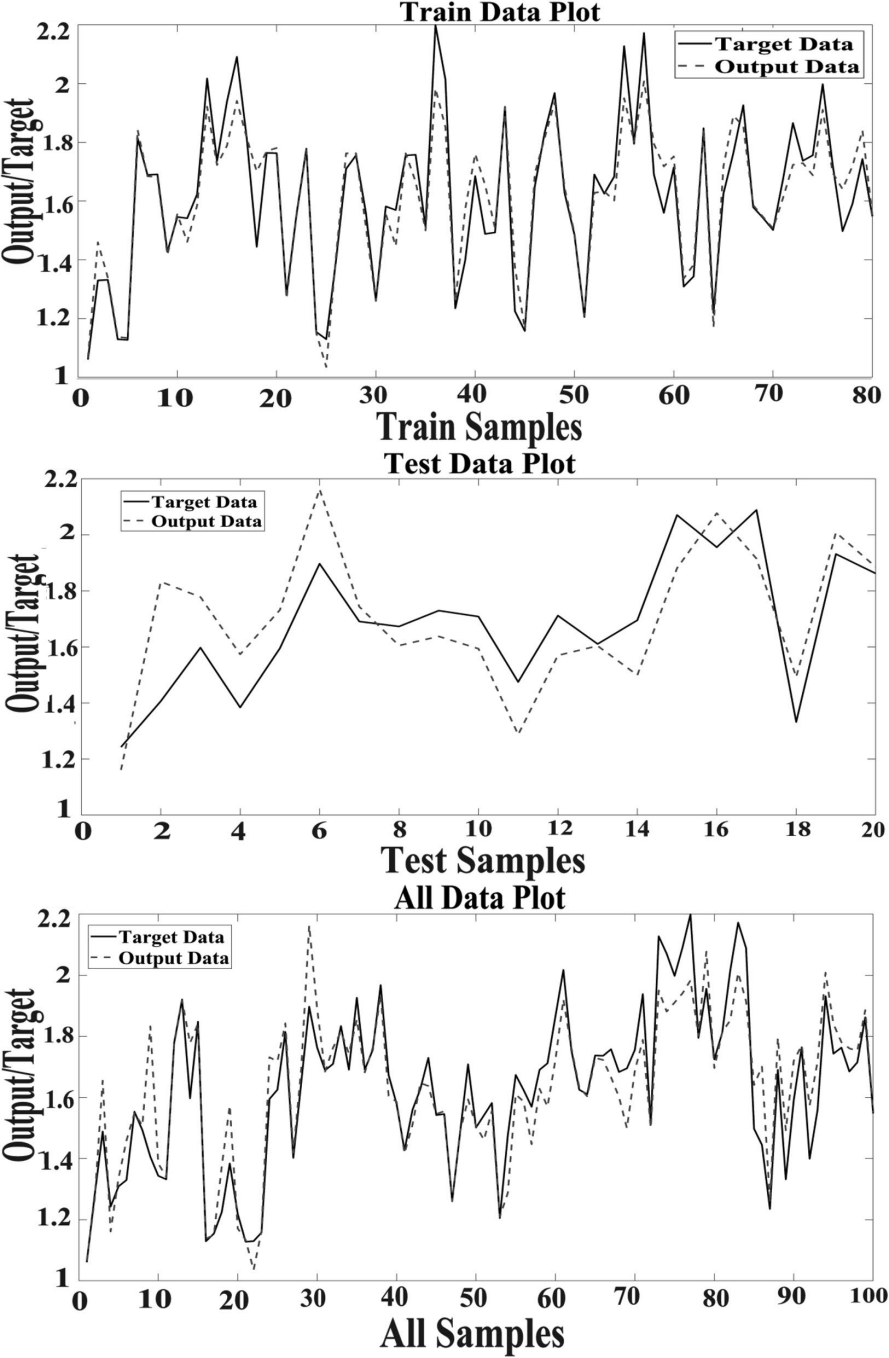
The outputs of SVM and target values of the hypericin content in data sets

In Fig. 7, there is a comparison between the simulated (output) values and the real target of SVM in datasets, which is evident and noticeable agreement between values.

### Model selection

**Fig. 8:**
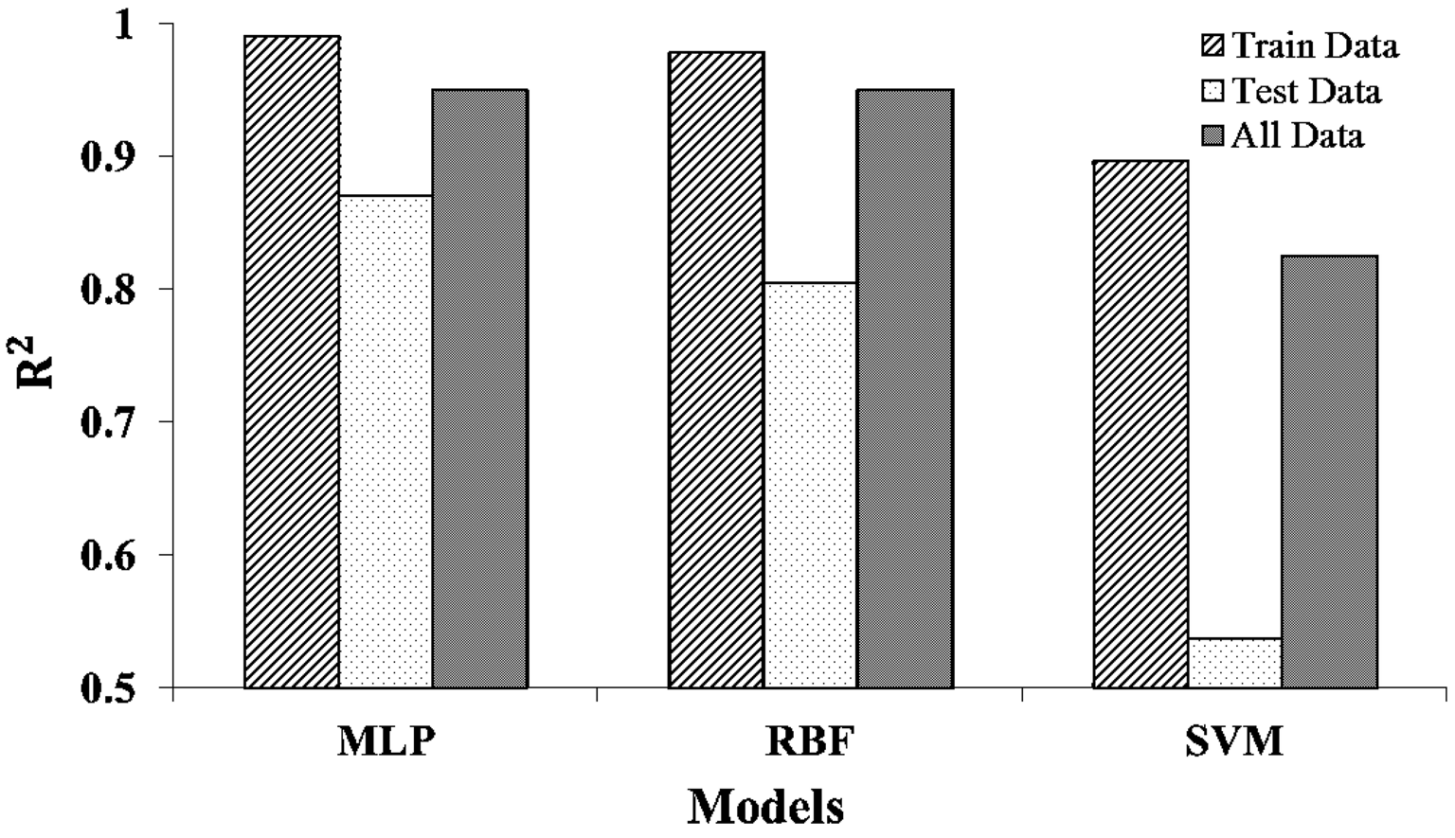
The performance measures of the MLP, RBFNN and SVM

The outputs of MLP, RBF, and SVM, have been compared in Fig. 8, demonstrating that MLP is the most suitable model to predict the content of hypericin. The MLP model showed the highest value of R^2^ in training, test, and all datasets when compared to RBFNN and SVM.

### Sensitivity analysis of MLP

**Fig. 9:**
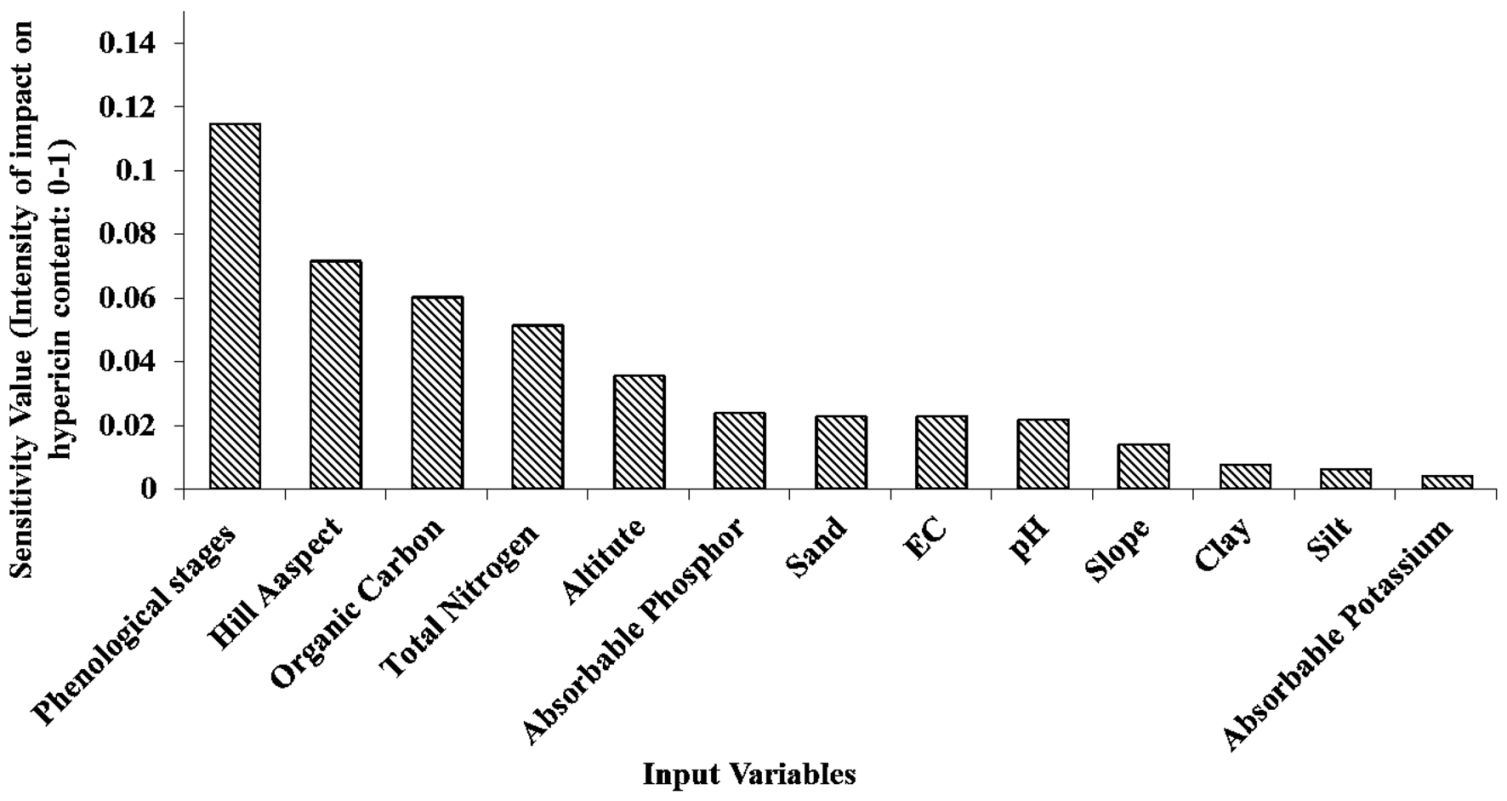
Sensitivity analysis of MLP model in hypericin content prediction

The MLP model sensitivities for input variables are shown in Fig. 9. It represents the standard deviations of MLP outputs (the content of hypericin) in response to each variable exhibited change. As we understand, the sensitivity value for each input variable is a value among zero to one which indicating standard deviations of hypericin content in response to input changes. As the changes in hypericin content in response to changes in an input variable increase, that variable is more significant in predicting hypericin content and the sensitivity value is closer to one.

Based on the results of sensitivity analysis, the values of the phenological stages, geographical hill aspects, organic carbon, total nitrogen and altitude were most significant inputs, influencing MLP outputs (Fig. 9). Other variables did not exhibit a significant effect on hypericin content changes in the model sensitivity analysis that could be due to the limited changes in some ecological variables values in the studied area (Table 4).

**Table 4:** The statistical results of variables quantity in *H. perforatum* samples

Average ± standard error (min, max)	Variable	Average ± standard error (min, max)	Variable
206.93 ± 5.54 (103, 287)	Absorbable potassium	2338.21 ± 75.33 (1103, 3822)	Altitude
3.11 ± 0.13 (0.5, 5.7)	Absorbable phosphor	19.5 ± 0.63 (5, 35)	Slope
0.34 ± 0.01 (0.1, 0.57)	Total nitrogen	2.63 ± 0.08 (1, 4)	Hill aspect
0.09 ± 0.003 (0.02, 0.2)	EC	0.85 ± 0.06 (0, 2.6)	Organic carbon
7.23 ± 0.05 (6.2, 7.9)	pH	42.5 ± 0.38 (35, 50)	Sand
1.99 ± 0.07 (1, 3)	Phenological stages	38.1 ± 0.61 (20, 50)	Silt
1.63 ± 0.02 (1.06, 2.2)	Hypericin content	19.4 ± 0.65 (5, 35)	Clay

**Fig. 10:**
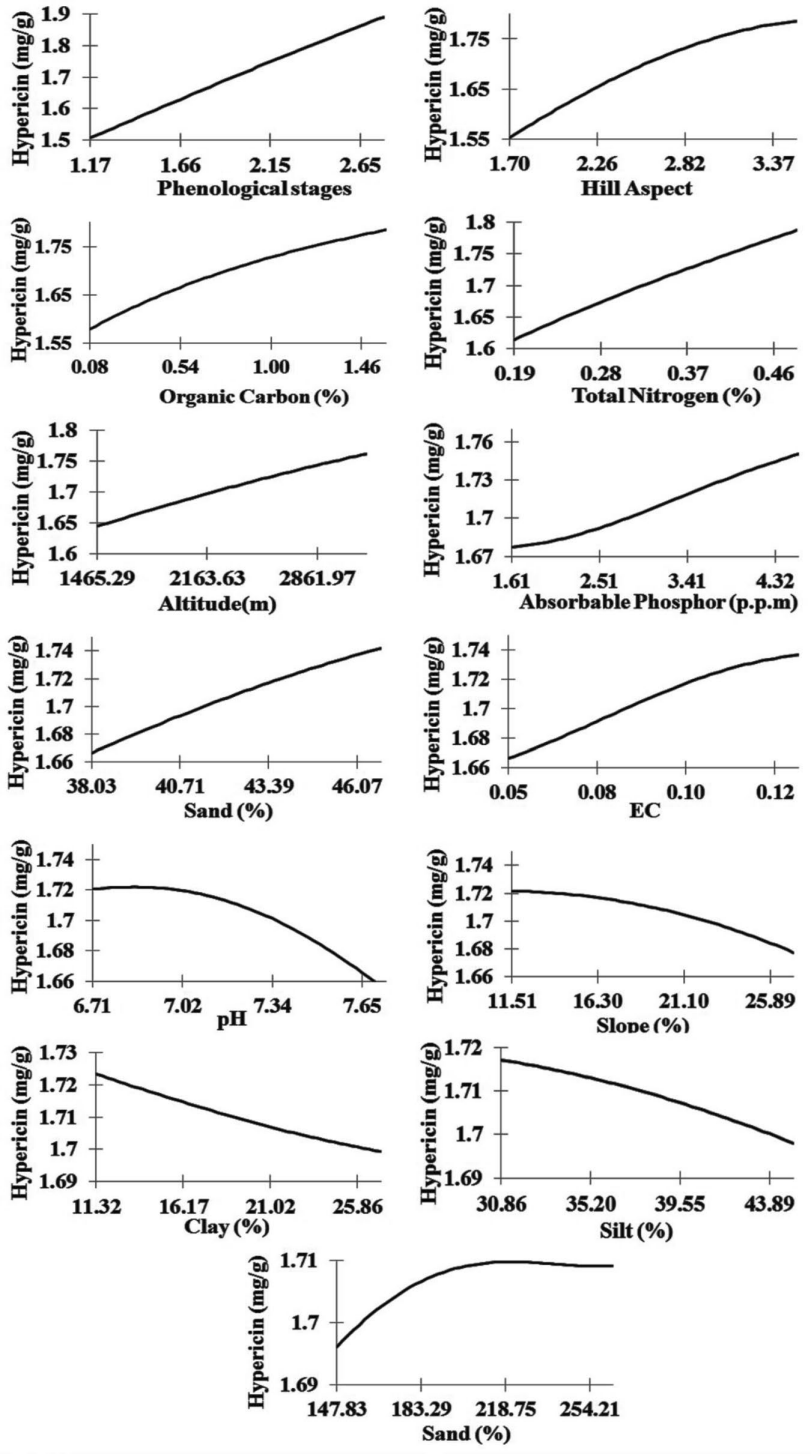
The trend of hypericin content changes using MLP output with varying the input variables

Considering trends in Fig. 10, phenological stages, hill aspects, organic carbon, total nitrogen and altitude are positively correlated to enhance hypericin content. So with the increase in the value of these variables, hypericin content followed a uniform and linear increasing trend. The phenological stages of the plant include the vegetative (1), flowering (2) and seed ripping (3) stage. In fact, the lowest content of hypericin is in the vegetative stage of the plants and increases in the flowering and seed ripping stages, respectively. Based on the results shown in Table 4, the range of changes in the percentage of total soil nitrogen in this study is 0.1 to 0.57. In Fig. 10, we find a linear increase in the amount of hypericin content with increasing the percentage of total soil nitrogen. The range of altitude changes in this study is between 1103 and 3822 m (Table 4). The main distribution of the *H. perforatum* in the study area is in this elevation range and with increasing altitude we see a linear increase in the amount of hypericin content (Fig. 10).

On the other hand, increasing the other two ecological variables, including the hill geographical aspect and soil organic carbon cause a nonlinear increase in hypericin content of samples. Of course, it should be considered that the changes in the hill aspects are from north (1) to east (2), south (3) and west (4). In fact, the lowest content of hypericin is found in plants grown on the northern slopes and increases on the eastern, southern and western slopes, respectively (Fig. 10). Since this relationship is nonlinear, not much difference is detected between the southern and western slopes. Based on the nonlinear relationship between soil organic carbon content and hypericin content in Fig. 10, the percentage of soil organic carbon, after crossing the 1.5 percent will not change the content of hypericin in samples significantly.

**Fig. 11:**
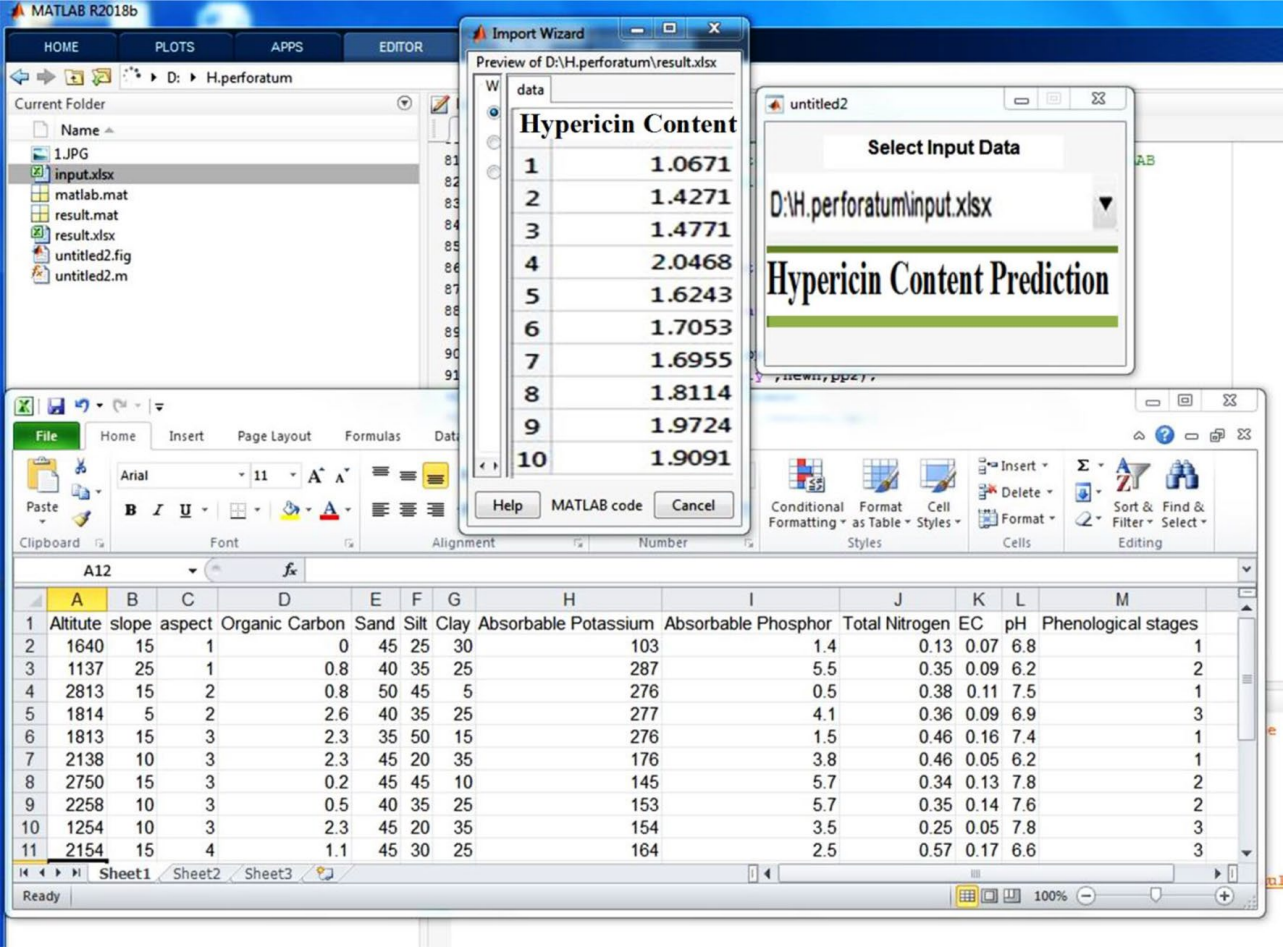
Hypericin content prediction based on ecological conditions of site and phonological stage

We also designed a new Graphical User Interface (GUI) for experts to use the MLP model in order to predict the content of hypericin in *H. perforatum* species. Indeed, the prediction of hypericin in *H. perforatum* under new ecological conditions of the site and the phonological stage is gaining popularity among users. GUI as an EDSS tool will be run on new data just by pushing the "Hypericin Content Prediction" button in Fig. 11 that illustrates the results of hypericin content prediction in 10 new samples based on the ecological conditions of the site and phenological stage.

## Discussion

Plant produced secondary metabolites play an important role in plant defense against various environmental stresses [15]. These secondary metabolites are extensively studied for their biomedicinal properties. The extraction of bioactive compounds from plants is vital in retaining its medicinal properties [15]. Ecological variables are mainly prominent not only in the growth of *H. perforatum* but also in the content of hypericin [8]. Various biotic and abiotic factors such as soil, developmental stage, ecological conditions, and altitude are correlated in the regulation of their biosynthesis and localization of secondary metabolites [43]. Due to medicinal uses of hypericin, we analyzed the association of different ecological conditions and altitudes with concentration of hypericin in *H. perforatum.*

The ANN modeling approach employed in this research not only utilizes the advantages of previously designed models [4, 15, 15] but also include new ANN technique. The ANNs have received greater attention in various applications because of its sensitivity, accuracy, non-destruction, and rapidity [42]. Mesgaran et al. [15] reported that ANN was most accurate method in predicting copper elements in the mineral areas compared to various methods such as fractal, principal components analysis, factor analysis. Similarly, Irmak et al. [10] investigated the prediction of soybean yield in varied temporal, soil and landscape situations using back-propagation neural network (BPNN) model and Tušek et al. [41] estimated total polyphenols content from chamomile, dandelion, marigold, and yarrow by using kinetic models. Out study demonstrated that the MLP model (R^2^ = 0.87 provided more suitable prediction of hypericin content in *H. perforatum* by utilizing the ecological condition of site, such as land form, soil characteristics and plant phenological stages in comparison to other models RBF (R^2^ = 0.8) and SVM (R^2^ = 0.54). We recorded minimum differences between observed and predicted values of hypericin in case of MLP. Since MLP is an environmental decision support system tool, it could be used to forecast the amount of hypericin in *Hypericum* in different habitats. Our findings suggest that using mathematical models such as MLP for predicting the amount of hypericin in *Hypericum* species could reduce time and cost required analytical methods. Savić et al. [15] reported suitability of MLP over central composite design (CCD) for analysis of total flavonoids in green tea. In another research, lettuce yield was calculated in the condition of water shortage by using the ANN technique and vegetation indices and revealed that ANNs best predicted lettuce yield with R^2^ values of 0.86, 0.75, and 0.92 for 100, 66, and 33% water treatments, respectively [18]. However, we compared three models MLP, RBF and SVM in our research and found that MLP was the most accurate model among them. These models have been recently compared in aesthetic quality prediction [13], chemical and medical sciences [15, 15] and vegetation density prediction [11] as well.

The results of sensitivity analysis revealed that, phenological stages, hill aspects, total N, altitude and organic C appeared the most fundamental factors significantly impacting hypericin content. Our observations regarding genotype, growing conditions, and developmental stage impacting hypericin content are in agreement with previous research [6, 43, 45]. The content of hypericin is varied based on different habitats and the stage of plant development also reported by Saddique et al. [15]. Previous research suggested that compounds in the *Hypericum* vary with climate and soil conditions which are consistent with our findings. Our results illustrated that there was a positive correlation between total N, organic carbon and the amount of hypericin. We observed that, as the total N and organic carbon increase in the soil, the hypericin increased accordingly. Yesaghi [44] confirmed that optimal conditions for the growth of *H. perforatum* depend on carbon and N-rich soil studying at three habitats in Golestan province, Iran. Moreover, our study indicated that the amount of hypericin improved from 1.65 to more than 1.75 mg/g (Fig. 10) by increasing altitude from 1000 to 4000 m above sea level which is in agreement with previous reports [43]. It is proposed that the combination of higher light intensity, UV-B irradiation and shortage of air temperature at the highest altitude could be a rational reason for increase in the hypericin content [3]. In terms of hill aspect, the highest content of hypericin was obtained from *H. perforatum* gathered in the western hills (1.8 mg/g). This result is similar to the study carried out by Zobayed et al. [45]. We studied the content of hypericin is influenced by ecological conditions such as altitude, soil factors and hill aspect which is in agreement to previous studies [1].

Computational simulation techniques and mathematical tools help us to predict the content of hypericin under ecological conditions [15]. Using the MLP model in the present study, we could predict the contents of hypericin in *H. perforatum* which will reduce the expensive and time-consuming phytochemical methods and laboratory experimentations. In addition, a designed Graphical User Interface (GUI) will help to run the MLP model on new data to easily discover the content of hypericin in *H. perforatum* by entering ecological conditions of site, land form, soil characteristics and plant phenological stages. These findings also could be applicable for rangeland managers, pharmacognosists, manufacturers and producers of medicinal plants, local beneficiaries and potentially various other field of studies.

## Conclusions

In this study, we predicted the content of hypericin in *Hypericum perforatum* using MLP, RBF and SVM models. These models were analyzed precisely with the aim of testing the most suitable model predicting hypericin content more accurately. Based on our observations, the MLP model was confirmed as superior compared to the other models considered in this study. Furthermore, we observed that phenological stages, hill aspects, organic carbon, total nitrogen and altitude have a positive impact on the content of hypericin. MATLAB 2018 software, will aid us to perform the model in the regions where the factors and tested variables in this research are in the range of studied area and also reduce time and costof experimental methods. Moreover, it can provide wider range of applicability to rangeland managers, pharmacognosists, manufacturers and producers of medicinal plants to specify habitats and plant individuals targeting the highest content of hypericin in a robust and repeatable way.

## Data Availability

The datasets used and/or analyzed during the current study are available from the corresponding author on reasonable request.
